# Evidence-Based Best Practices for Diabetes Education and Self-Care in Appalachia: A Literature Review

**DOI:** 10.13023/jah.0703.10

**Published:** 2025-09-01

**Authors:** Angela J. Occidental, Inga M. Zadvinskis, Jacqueline Hoying

**Affiliations:** Duncan Medical Family Practice; OhioHealth Riverside Methodist Hospital; The Ohio State University College of Nursing

**Keywords:** Appalachian region, diabetes, patient education

## Abstract

**Introduction:**

The Appalachian Region has a higher prevalence of type 2 diabetes mellitus (T2DM) and associated adverse health outcomes. Although numerous papers report best practices for diabetes care and education, a clinician-friendly, synthesized summary of best practices tailored to Appalachian cultural preferences is lacking.

**Purpose:**

This paper uses the Melnyk & Fineout-Overholt evidence-based framework to identify the best practices for T2DM care and education for Appalachian residents.

**Methods:**

A comprehensive literature search using the databases CINAHL, Academic Search Complete, and PubMed was guided by a PICOT question. Quality appraisals were completed for fifty-two articles; thirty papers were selected to synthesize best practices, delivery methods, lifestyle modifications, and outcomes.

**Results:**

Best practices for T2DM education are recognizing how culture and the social determinants of health (SDOH) influence care, using a multidisciplinary team, and screening for diabetes knowledge and distress. Beneficial education topics are nutrition, weight management, medication management, stress management, health maintenance screenings, and lifestyle modification including exercise. Access to care may be increased by using digital or online formats.

**Implications:**

T2DM is a complex chronic health issue; strategies are needed to address health disparities and SDOH in Appalachia. Future research is needed to determine the best practices for the duration and frequency of diabetes education and to determine ways to engage residents in diabetes care. Partnerships with local organizations may create a support network for diabetes management. Decision-makers can use these best practices to pursue interventions that engage the Appalachian community in better diabetes care.

## INTRODUCTION

Effective diabetes management is essential for preventing health complications and containing healthcare costs. Diabetes mellitus (DM) is a chronic endocrine disorder that affects glucose metabolism, causing hyperglycemia. Type 1 diabetes occurs when the body does not produce enough insulin. In contrast, Type 2 DM (T2DM) is due to insulin resistance and is often related to lifestyle factors like lack of physical activity/exercise, diet, and being overweight. Unmanaged diabetes may lead to multiple adverse health outcomes such as renal failure, limb amputations, blindness, and increased mortality from cardiovascular disease.^1^ Fortunately, T2DM can be managed with medication, diet, and lifestyle interventions, but creating healthy habits and implementing lifestyle changes can be challenging.

In the Appalachian Region, numerous health disparities and social determinants of health (SDOH)^1^,^2^ influence diabetes care. Specifically, barriers affecting diabetes education and care in Appalachia include transportation issues,^3^–^5^ low health literacy,^3^,^6^ food insecurity,^7^–^9^ and financial constraints.^6^,^9^,^10^ Additionally, cultural factors significantly impact diabetes self-management. Appalachian culture strongly emphasizes self-sufficiency, which may lead individuals to try self-treatment or home remedies before seeking help from a healthcare provider.^11^ Reluctance to seek medical advice can prevent Appalachian residents with diabetes from obtaining the essential education and support needed to improve their diabetes self-management skills.^12^ Integrating culturally relevant best practices into diabetes education and care is vital for increasing engagement in diabetes self-management, promoting better health outcomes, improving glucose control, and preventing the development of adverse health outcomes related to unmanaged diabetes, such as those stated above.

### Organizational Assessment and Context of the EBP Initiative

Scioto County, Ohio, is ranked as the unhealthiest county in Ohio,^13^ with contributing factors such as higher levels of cancer, heart disease, lung disease, and accidents.^13^ The diabetes prevalence rate in this county is 13%, compared with the national average of 10%.^13^ Numerous SDOHs influence health care in Scioto County, including high rates of unemployment, widespread opioid addiction, and below-average household income.^13^ Access to health care provided by diabetes specialists is particularly problematic in Scioto County. Diabetes education is available by referral, and one endocrinology practice offers video-based physician visits and in-person care with nurse practitioners. However, patients may resist seeking specialty care due to the higher costs and transportation issues.

## METHODS

Evidence-based practice (EBP) is a problem-solving approach to clinical decision-making that combines the best available evidence, clinician expertise, and patient preferences.^14^ The seven-step EBP process consists of step 0 (spirit of inquiry), step 1 (clinical question), step 2 (evidence search), step 3 (evidence appraisal and synthesis), step 4 (evidence integration), step 5 (evaluation), and step 6 (dissemination).^14^ This literature review aims to identify best practices for T2DM and education specific to Appalachian residents for a practice change using the Melnyk & Fineout-Overholt seven-step model for EBP.^14^ This model was chosen because it includes an easy-to-follow EBP process, focuses on developing EBP mentors, and provides a roadmap to create an organizational change to improve patient outcomes.

### The Seven Steps of EBP

#### Step 0: Clinical inquiry

##### Problem identification

The clinic was not achieving the Centers for Medicare and Medicaid Services (CMS) quality measure for diabetes poor control (hemoglobin a1c (HbA1c) > 9.0%).^15^ In 2021, the APRN (Advanced Practice Registered Nurse) implemented a project focusing on diabetes diet education in the primary care clinic. Initially, HbA1c values improved, but these improvements were not sustained. Multiple patients experienced cardiac issues, strokes, kidney disease, and eye problems related to diabetes. The APRN learned that people did not understand how diabetes could affect their health. Numerous patients expressed that they felt that “having sugar” (i.e., elevated blood sugar levels) was just a part of life, and they did not feel as though anything could be done to improve. Overall, patients were resistant to attending formal diabetes education due to cost, time, and transportation issues, and they wanted to attend only one appointment. This discovery sparked a spirit of inquiry, prompting the APRN to explore whether there were published evidence-based best practices for diabetes education specific to the Appalachian population. Facilitators for an EBP initiative to provide better diabetes care are leadership support, financial incentives for meeting the CMS quality measure for HbA1c,^15^ and potentially improved health outcomes.

#### Step 1: PICOT question

A PICOT question directed the literature search. PICOT stands for Population, Intervention, Comparison, Outcome, and Time. Our PICOT question was: in persons with diabetes in Appalachia (P), how does diabetes education recognizing the social determinants of health (I) compared with usual care (C) affect outcomes (O)? The underlined words highlight key search terms. Time (T) was not included as a search term to keep the inquiry broad and to avoid time restrictions for measured outcomes.

#### Step 2: Literature search

The literature search was conducted from October 2023 – January 2024 in the databases CINAHL, Academic Search Complete, PubMed, and Cochrane. Limits included the English language. No limits were placed on the publication date. Search terms were:

“diabetes” AND “Appalachia” **(81 results)**

“diabetes” AND “social determinants of health or SDOH” **(1951 results)**

“diabetes” AND “SDOH” AND “rural” **(65 results)**

“diabetes education” AND “rural” **(908 results)**

“diabetes” AND “rural” AND “HbA1c” **(304 results)**

“diabetes” AND “Appalachia” AND “HbA1c” **(6 results)**

“diabetes education” AND “rural or Appalachia” AND “A1c” **(62 results)**

“diabetes” AND “Appalachia” **(328 results)**

“diabetes” AND “patient education” AND “Appalachia” **(1011 results)**

“Appalachia” AND “diabetes education” AND “outcomes” **(680 results)**

The primary author screened abstracts of the articles, seeking matches with population (adult patients with T2DM), setting (rural or Appalachian), intervention (outpatient diabetes care or education), and outcomes (clinical, such as HbA1c changes, or self-management skills and knowledge).

The primary author also performed study selection and quality appraisals as part of a post-doctoral program with guidance from the faculty mentors. Disagreements regarding study selection and assessment for risk of bias were resolved through discussion, mentorship, and consensus. Screening revealed 187 articles as potentially relevant. After reviewing the abstracts, articles were excluded due to being duplicates (39 results), not aligning with the clinical question (81 results), and being unavailable as full text (15 results). The remaining 52 articles were selected for critical appraisal.

#### Step 3: Critical appraisal and synthesis of articles

The Helene Fuld Health Trust National Institute for Evidence-based Practice in Nursing and Healthcare rapid critical appraisal tools^16^ were used to appraise the 52 articles. Studies were eliminated for incorrect setting (inpatient) (four papers), focus on children or adults with type 1 diabetes (eight papers), programs based on diabetes prevention (not management) (seven papers), or gestational diabetes focus (three papers). Multiple synthesis tables were created to categorize themes found in the literature, including conceptual frameworks and models, best practices, delivery methods, lifestyle modifications, and outcomes. After excluding studies that did not apply to the desired setting or patient population, the final review included 30 articles.

##### Levels of evidence

The Melnyk and Fineout-Overholt seven-level evidence hierarchy was used to categorize the strength of the literature.^14^ In this hierarchy, the most rigorous evidence is from meta-analyses and systematic reviews, followed by randomized controlled trials and other research designs, with expert opinion at the lowest level. The body of literature included randomized controlled trials (two articles), clinical practice guidelines (two articles, both based on systematic reviews), cohort studies (five articles), systematic reviews of descriptive studies (four articles), descriptive studies (16 articles), and an expert opinion paper (one article).

##### Conceptual frameworks and models for diabetes care

Conceptual frameworks and models can guide initiatives by defining and organizing key concepts. In this body of literature, 11 papers used ten frameworks/models; two papers used the chronic care model.^2^,^17^ The 2024 American Diabetes Association guidelines recommended aligning approaches to diabetes management with the Chronic Care Model.^2^ This model emphasizes a person-centered care team and ongoing collaboration between team members.

##### Best practices for diabetes education in a rural population

The most frequently cited best practices for diabetes education and care in Appalachia were understanding how cultural norms and SDOH affect care,^2^,^5^,^12^,^18^,^19^ using a multidisciplinary team,^2^,^8^,^18^,^20^–^23^ involving community health workers and family support,^2^,^5^,^12^,^19^,^20^,^23^,^24^ screening for diabetes knowledge,^5^,^19^–^23^,^25^,^26^ diabetes distress,^2^,^5^,^19^ and expanding access to care digitally or online.^2^,^7^,^8^,^18^,^20^,^21^,^24^–^26^ (Table 1). Initiatives with a multidisciplinary team (including pharmacists, community health workers [CHWs], dieticians, nurses, physicians, and nurse practitioners) showed improvement in self-care measures, including diabetes knowledge, increased satisfaction with diabetes care, and sustained reductions in HbA1c.^18^,^21^,^23^,^27^ Multidisciplinary care teams in Appalachia have included CHWs.^20^,^24^,^28^ CHWs partner with healthcare providers to provide evidence-based, cost-effective care in underserved communities.^2^

The American Diabetes Association advised screening for diabetes distress at least annually.^18^ Persons experiencing diabetes distress feared developing diabetes-related complications yet disengaged from self-care activities and exhibited low diabetes knowledge.^9^

##### Frequency and delivery method

The evidence did not suggest a best practice for the duration or frequency of diabetes education sessions. The most significant HbA1c reductions were noted in diabetes self-management education (DSME) programs consisting of ten or more hours.^12^,^18^ Interventions with more patient contact hours and programs based on theoretical models demonstrated improved outcomes.^20^ A 2015 systematic literature review noted that five theory-based studies demonstrated improved glycemic control, and the authors surmised that the reasons for improvement were because theory-based interventions focused on behavior changes.^20^

The CMS will reimburse for ten hours of diabetes education at diagnosis, and two hours each subsequent year for Medicare Part B beneficiaries.^12^ However, criteria must be met before DSME services are reimbursed.^18^ These three criteria include: (1) who can deliver the education (dietician, diabetes educator), (2) location (in-person or telehealth), and (3) delivery method (group or individual).^18^ Providers must obtain and maintain certification that meets the National Standards for Diabetes Self-Management Education and Support (DSMES) through a CMS accredited organization to receive reimbursement for DSME.^18^

Additionally, patients must pay copayments for DSME services. These criteria could limit Appalachian residents’ access to DSME, as dieticians and certified diabetes educators may not be locally accessible, and paying additional fees may not be achievable. Diabetes education delivered by telemedicine, digital, or online format effectively increased availability and showed reduced HbA1c.^2^,^20^,^29^

##### Components and lifestyle modifications for diabetes education

Nutrition education and physical activity were frequently cited lifestyle modifications.^4^,^5^,^8^,^26^,^30^,^31^ Components of nutrition education included reading food labels,^12^,^19^,^21^,^26^ carbohydrate counting,^5^,^12^,^18^,^26^ and choosing healthy foods with limited finances.^2^,^21^,^32^ Researchers often used the plate method, a visual representation of food portioned on a plate.^2^,^21^,^26^,^30^ Four programs centered on increased vegetable consumption.^7^,^8^,^30^,^31^ When providing diet education, experts recommended customizing meal plans while considering sociocultural factors,^33^ health literacy,^2^,^6^,^30^ and dietary knowledge.^18^,^33^

Lifestyle modification components included exercise,^5^,^12^,^18^,^26^,^31^ health maintenance screenings,^5^,^12^,^21^,^22^,^26^,^31^ stress management,^5^,^12^,^18^,^19^,^21^–^23^ medication management,^5^,^12^,^21^,^22^,^31^ and weight management.^8^,^12^,^18^,^34^ (Table 2). The ADA recommends 150 minutes weekly or 30 minutes of exercise on most days.^18^ Carpenter discovered that Appalachian residents often did not meet the recommended exercise goal^35^ (Table 3).

## RESULTS

Evidence-based diabetes care positively impacted HbA1c. In a meta-analysis of 13 studies, Lepard et al. determined that three studies had a statistically significant reduction in HbA1c (8.2% to 7.1%; 9.4% to 8.2%; 7.35% to 6.97%).^20^ Five studies demonstrated HbA1c reductions in both the control and the intervention groups, but since there were no statistically significant changes between the groups, the researchers questioned whether the intervention led to the HbA1c reduction. Five studies did not show statistically significant reductions in HbA1c, but even slight reductions could be clinically significant. Studies with extended follow-up (i.e., beyond six months), telehealth, online components, and collaboration with family or CHW demonstrated sustained, statistically significant differences after 18 months.^20^

A community-based study with collaboration between healthcare providers and community revealed an HbA1c reduction of 24.7% over 12 months.^12^ A non-randomized prospective study utilizing a multidisciplinary team showed an initial decline of 2.0% in HbA1c in the intervention group and 0.9% in the comparison group.^21^ A study using an interdisciplinary team in an Appalachian primary care clinic saw a 22% decrease in HbA1c after six months, and the results were sustained at 18 months 22 Lastly, a study using CHWs to provide diabetes education in a rural Appalachian population demonstrated a decrease in HbA1c from a mean of 7.76% to 7.42%.^23^

Participation in self-care activities increased in three studies and did not change in one study,^20^,^24^,^29^ and physical activity increased in two studies.^20^,^23^ Weight appeared more challenging to change; while it decreased in three studies,^12^,^20^ five studies reported no change in weight.^20^,^23^ Decreased body mass index (BMI) was noted in two studies^12^,^20^ while two other studies found no change in BMI.^22^,^23^ Decreased blood pressure (BP) was noted in one article,^12^ another study reported a partial decrease in BP, and four studies reported no change.^20^,^34^ Triglycerides and low-density lipoprotein (LDL) cholesterol were decreased in two studies^20^; five reported no change^20^,^22^, and one reported no change to the total cholesterol values.^20^,^22^ Healthcare engagement improved in four studies and did not change in one study 12,20 (Table 4).

## IMPLICATIONS

Suggested actions for health professionals caring for people with T2DM living in Appalachia include recognizing how culture and SDOH influence diabetes care. Appalachian residents often prefer to have rapport and a period of getting to know their healthcare provider rather than being told what to do by someone who may not understand their circumstances.^5^

Many harmful stereotypes have been attributed to Appalachian residents; patients may feel judged by healthcare providers, resulting in them avoiding seeking healthcare services until health problems are severe.^36^ A strong desire for independence and reluctance to ask for help are common Appalachian cultural traits; however, healthcare providers may perceive these characteristics as a person being obstinate or unintelligent.^11^ Health professionals must learn about Appalachian cultural values and practice cultural humility when interacting with people from this region.^11^ Involving family is a key strategy to address challenges related to diabetes management. Lifestyle changes necessary for diabetes management are more likely to succeed if the family acknowledges and supports the need for change.

Healthcare providers should also assess diabetes knowledge and distress, provide care in a digital or online format, and use a multidisciplinary team. Offer education, including nutrition, lifestyle modification, and assessment of diabetes knowledge and distress. Anticipated outcomes from these best practices for Appalachian residents are decreased HbA1c, improved diabetes knowledge, decreased diabetes distress, enhanced patient engagement, and potentially increased reimbursement for diabetes care. To enhance diabetes care in the clinic, the primary author is implementing evidence-based recommendations, including screening for diabetes knowledge and diabetes distress, and offering online education. Barriers include technological literacy and internet access in the region. The team will evaluate short-term (quarterly) and long-term outcomes for sustainability with plans to disseminate results.

This literature review is limited by potential article selection bias. However, the authors have conducted critical appraisals and provided synthesis tables that supplement the narrative summary, allowing the reader to review the findings in an objective, clinician-friendly, easily understandable format.

## CONCLUSION

Diabetes is a complex chronic health issue. Appalachian communities need strategies to address health disparities and SDOH. Researchers may consider exploring best practices for the duration and frequency of diabetes education and determining ways to motivate Appalachian residents to engage in lifestyle changes like exercise. Partnerships with local organizations, such as schools or faith-based organizations, may create a support network for diabetes management. Decision-makers can use these best practices to pursue interventions that engage the Appalachian community in better diabetes care.

**SUMMARY BOX**

**What is already known about this topic?**

The prevalence rate of diabetes is higher in the Appalachian Region; however, barriers such as geographic access, socioeconomic factors, and health literacy affect diabetes self-management practices.

**What is added by this report?**

Identifying the evidence-based best practices for diabetes education can help healthcare providers and decision-makers focus on interventions that will improve population health.

**What are the implications for future research?**

Research is needed to test the duration and frequency of diabetes education for the Appalachian population; determine impact of implementing evidence-based, tailored best practices on long-term clinical outcomes; and find ways to engage Appalachian residents to make lifestyle changes.

## Figures and Tables

**Table 1:** Best Practices for Diabetes Education and Care in an Appalachian Population

Best Practices	Citation Number (See Notes)												
	2	7	8	12	18	19	20	21	22	23	24	25	26
Consider SDOH	X	X		X	X	X		X	X	X			
Incorporate telemedicine or online healthcare	X^a^	X	X		X		X	X			X	X	X
Assess diabetes knowledge						X	X	X	X	X		X	X
Use an interdisciplinary team	X		X	X			X	X	X	X			
Provide culturally sensitive care	X				X	X	X			X			
Use community health workers/lay educators	X			X	X	X	X^b^			X	X		
Provide group-based education				X		X		X				X	
Screen for diabetes distress	X					X							
Follow-up and reinforce education							X	X					
Consider health literacy					X	X							

NOTES:

a = technology includes continuous glucose monitoring systems

b = included family and peer support 2. American Diabetes Association Professional Practice Committee (b) (2024) 7. Harris et al. (2022) 8. Sastre LR, et al. (2023) 12. Freeman K, et al. (2018) 18. American Diabetes Association Professional Practice Committee (a) (2024) 19. Misra & Fitch (2020) 20. Lepard MG, et al. (2015) 21. Jessee BT & Rutledge CM (2012) 22. King DE et al. (2019) 23. Feltner F, et al. (2017) 24. Marsh Z, et al. (2021) 25. Ladner KA, et al. (2022) 26. Hunt CW, et al. (2018).

**Table 2:** Diabetes Educational Components/Content for an Appalachian Population

Citation Number (See Notes)							

Education Component/Content	12	18	19	21	22	23	26

Nutrition education	X	X^a^	X	X	X		X

Stress and coping techniques	X	X	X		X	X	X
Medication management	X	X		X	X		X
Carbohydrate counting	X	X		X			X

Reading food labels	X		X		X		X

Screenings Foot care/eye exam				X	X	X	

Plate method	X	X		X			

Weight management	X	X					X

Healthy eating on a budget		X	X		X		

Fast food alternatives	X				X		

NOTES:

a = discussed balance of fats, carbohydrates, sodium, and alcohol use 12. Freeman, K et al. (2018) 18. American Diabetes Association Professional Practice Committee (a). (2024) 19. Misra R & Fitch C (2020) 21. Jessee BT & Rutledge CM (2012) 22. King DE, et al. (2019) 23. Feltner, F et al. (2017) 26. Hunt CW, et al. (2018).

**Table 3:** Lifestyle modifications for diabetes management in an Appalachian Population

Lifestyle Modification	Citation Number (See Notes)						
	8	12	18	21	22	26	31
Nutrition education c	x	x	x	x	x	x	x
Self-care activities a		x		x	x	x^b^	x
Exercise/physical activity		x	x			x	x
Medication management		x		x	x		x
Stress management d		x	x	x	x		
Weight management	x	x	x		x		

NOTES:

a = self-care activities include home glucose monitoring, foot care, retinal eye exams, smoking cessation, avoiding alcohol, and vaccinations

b = included insulin administration

c = included carbohydrate counting, the plate method plan, healthy substitutions at restaurants, increasing fruit and vegetable intake, and eating healthy on a budget

d = includes diabetes distress 8. Sastre LR, et al. (2023) 12. Freeman, K et al. (2018) 18. American Diabetes Association Professional Practice Committee (a). (2024) 21. Jessee BT, et al. (2012) 22. King DE, et al. (2019) 26. Hunt CW, et al. (2018) 31. Rafie C et al. (2021)

**Table 4:** Outcomes of Diabetes Education Interventions in an Appalachian population.

Outcomes	Citation Number (See Notes)					
	12	20	21	22	23	26
Hemoglobin A1c	↓	↓ (3)	↓	↓	↓	
		↘ (5)				
		↓→ (5)				

Diabetes knowledge		↑ (4)	↑	↑	↑	↑
		↖ (1)^c^				

Self-management behaviors		↑ (1) *	↑		↑	←→

BMI	↓	↓ (1)		←→	←→	

Weight	↓	↓ (2)			←→	
		←→ (5)				

Blood Pressure		↓ (1)		←→		
		↘ (1)				
		←→ (3)				

Triglycerides, LDL Total cholesterol^b^		↓ (2)		←→		
		LDL		←→^b^		
		←→ (4)				

Physical activity		↑ (1)			↑	

Engagement in healthcare	↑	↑ (3)				
		←→ (1)				

NOTES:

Shaded cells indicate desirable outcomes.

Systematic Review (#) = the number of studies that reported the outcome

* =glucose monitoring

a = diet, exercise, foot care, blood sugar monitoring and management, and medication use

b = discussed total cholesterol

c = knowledge increased with the number of sessions ↑ = increase; ↓ = decrease; ↓→= decrease not statistically significant; ←→ = no change; ↖ = partial increase; ↘ = partial decrease 12. Freeman K et al.(2018) 20. Lepard MG, et al.(2015) 21. Jessee BT, & Rutledge CM (2012) 22. King DE et al.(2019) 23. Feltner F, et al.(2017) 26. Hunt CW, et al. (2018).
